# New Viscoelastic Hydrogel Hymovis MO.RE. Single Intra-articular Injection for the Treatment of Knee Osteoarthritis in Sportsmen: Safety and Efficacy Study Results

**DOI:** 10.3389/fphar.2021.673988

**Published:** 2021-05-28

**Authors:** Andrea Bernetti, Francesco Agostini, Federica Alviti, Nicola Giordan, Federica Martella, Valter Santilli, Marco Paoloni, Massimiliano Mangone

**Affiliations:** ^1^ Department of Anatomical and Histological Sciences, Legal Medicine and Orthopedics, Sapienza University of Rome, Rome, Italy; ^2^ Fidia Farmaceutici S.p.A, Abano Terme, Italy; ^3^ Local Health Authority of Latina, Latina, Italy

**Keywords:** sport, overuse injury, Knee, viscosupplementation, hyaluronic acid, osteoarthritis

## Abstract

Viscosupplementation by hyaluronic acid (HA) is recommended for non-surgical management of knee osteoarthritis (OA). This study investigated the efficacy and safety of a single i.a. (32 mg/4 ml) Hymovis MO.RE. injection, a new HA derivative hydrogel, for the treatment of adult regular sports players affected by knee OA arising from overuse injuries. Patients were prospectively enrolled if regularly practicing sports and diagnosed with Kellgren-Lawrence grade I-III OA. They received a single Hymovis MO.RE. intra-articular (i.a.) injection and were evaluated 30, 90, 180, and 360 days thereafter. The assessment involved measuring changes in knee function, pain, the activity of daily living (ADL), and quality of life (QOL) by using the Knee injury and Osteoarthritis Outcome Score (KOOS), GAIT analysis, the Western Ontario and McMaster Universities Osteoarthritis Index (WOMAC) scores for knee pain (WOMAC A) and function (WOMAC C), and a visual analogue scale (VAS) pain score. The study involved thirty-one patients, 23 women and eight men, whose median age was 49. KOOS function subscore, as well as GAIT cadence and velocity, showed a statistically significant increase at each time-point after injection (*p* < 0.0001). WOMAC, KOOS pain, symptoms, ADL, and QOL scores also significantly improved at all control visits. No severe adverse events or treatment-related events were detected. A single Hymovis MO.RE. (32 mg/4 ml) intra-articular injection provides a rapid, lasting, and safe response in regular sports players affected by knee OA, possibly representing a viable therapeutic option for this demanding patient subgroup. Further investigations are necessary to confirm these findings.

## Introduction

Frequent or intense stress can cause cartilage wear and inflammation, resulting in overuse injuries (Aicale et al., 2018). These are widespread in active people and athletes, accounting for 30% of all injuries related to sports activities (Taunton et al., 2003; Yang et al., 2012; Bernetti et al., 2014). The incidence of these injuries peaks in people younger than 30 (Majewski et al., 2006). About one third of overuse injuries involve the knee joint (Majewski et al., 2006; Gage et al., 2012; Yang et al., 2012; Bernetti et al., 2014). An evaluation of osteoarthritis in the early stage is therefore essential to evaluate the most suitable treatment (Iolascon et al., 2017; Luyten et al., 2018; de Sire et al., 2020; de Sire et al., 2021).

Inadequate recovery time from knee overuse injuries and joint overload are typical in sports professionals and lead to cartilage degeneration, causing knee osteoarthritis (OA) (Vannini et al., 2016; Yunus et al., 2020). Knee OA has been observed to affect former amateurs and sports professionals more than control groups who did not perform sports activities regularly (Klünder et al., 1980; Drawer and Fuller., 2001). The main symptoms of knee OA are joint pain and loss of function (Dantas et al., 2020), resulting in reduced performance or early retirement from sport (Drawer and Fuller., 2001; Vannini et al., 2016). The first-line treatment of overuse knee OA in active people and sportsmen is non-surgical (Leslie, 2000; Michael et al., 2010; Collins et al., 2019; Bernetti et al., 2020; Masiero et al., 2020). Conservative, non-surgical approaches aimed to delay knee arthroplasty may involve pharmacological regimens, including acetaminophen, NSAIDs, and intra-articular (i.a.) injections, as well as non-pharmacological interventions such as weight loss and patient education (Paoloni et al., 2015; Gress et al., 2020; Arden et al., 2021). Among conservative approaches, viscosupplementation (VS) through Hyaluronic acid (HA) i. a. injection may represent a viable option for people who regularly practice sport diagnosed with knee OA. HA, which is the main constituent of synovial fluid, provides shock absorption, joint lubrification, and chondroprotection (de Campos et al., 2019). The biological effects of HA viscosupplementation include moderate anti-inflammatory and anti-oxidant action, cytokine-induced enzyme reduction, anabolizing effect on cartilage, and pain relief by masking joint nociceptors (Waddell and Bert, 2010; de Campos et al., 2019; Henrotin et al., 2019).

VS through i.a. HA injection is recommended in the management of mild to moderate knee OA, given its efficacy in providing pain relief in symptomatic patients (Bruyère et al., 2014; Trojian et al., 2016; Bannuru et al., 2019; Concoff et al., 2019; Henrotin et al., 2019; Arden et al., 2021). HA VS may also be a viable option for patients which may have contraindications of using NSAIDs or which do not respond to NSAIDs treatment, as recommended by the current European Society for Clinical and Economic Aspects of Osteoporosis, Osteoarthritis and Musculoskeletal Diseases (ESCEO) and Osteoarthritis Research Society International (OARSI) guidelines (Bannuru et al., 2019; Arden et al., 2021).

Hymovis MO.RE. (Mobile Reticulum) (a CE-marked Class III Medical Device, Fidia Farmaceutici SpA, Italy), is a new viscoelastic hydrogel obtained from the HYADD®4 HA derivative (Finelli et al., 2009; Bernetti et al., 2020). Hymovis MO.RE. linear polymers are stabilized by hydrophobic and hydrophilic interactions, forming a mobile reticulum (MO.RE. Technology) that recovers its original structure even after repeated mechanical stress (Finelli et al., 2011; Santilli et al., 2018). These features make sportsmen and people performing recreational activities the ideal study target to investigate Hymovis MO.RE. efficacy. From the point of view of the characteristics of MO.RE. hyaluronic acid, it presents peculiar differences compared to other hyaluronic acids used for intra-articular infiltrative therapy. Particularly, it is interesting to underline how its molecular form, consisting of polymeric chains of hyaluronic acid stabilized by reversible hydrophobic and hydrophilic bonds, makes it behave from the physical point of view as a high molecular weight hyaluronic acid, and from the point of view of the biological as a low molecular weight hyaluronic acid, with the possibility of interacting with the CD34 membrane receptors and restoring joint homeostasis (Finelli et al., 2011; Santilli et al., 2018).

Clinical investigations in cohorts of non-target patients treated by Hymovis VS have already been performed, showing positive results. A retrospective study, designed to evaluate the clinical efficacy of two intra-articular Hymovis infiltrations (24 mg/3 ml) administered one week apart in patients affected by mild-to-moderate knee OA (Kellgren-Lawrence grades II-III), demonstrated that Hymovis is an effective treatment due to its protective and reparative effects on the synovial fluid of the knee joint. This treatment schema reduced Western Ontario and McMaster Universities Osteoarthritis Index (WOMAC) scores and NSAIDs/acetaminophen consumption for at least 6 months (Priano, 2017). Benazzo et al. (2016) demonstrated that two i.a. Hymovis (24 mg/3 ml) injections one week apart alleviated knee pain since the first treatment cycle and that patients treated with two cycles of i.a. injections of Hymovis experienced progressive pain reduction maintained for 1 year after the last injection. A recently published prospective randomized investigation by Pavelka et al., comparing three different HA-formulations in a 60-patient study, demonstrated that a single injection of Hymovis (32 mg/4 ml) also called Hymovis MO.RE., was safe and effective as a single injection of Hymovis (48 mg/6 ml) or Synvisc-One (48 mg/6 ml). Based on this study, Hymovis MO.RE. (32 mg/4 ml) was identified as the preferable dosage for treating symptomatic knee OA by a single i.a. injection (Pavelka et al., 2020). Moreover, a single Hymovis MO.RE. (32 mg/4 ml) injection enabled a significant decrease in WOMAC-A pain and WOMAC-C function scores at day 180 after treatment in patients older than 60 years of age and affected by mild to moderate knee OA.

Sports players expect any knee OA treatment to lead to a rapid improvement of functional limitations and long-lasting symptom relief, allowing early return to sports activities. They may therefore show reduced compliance to long-term therapies. Treating them through a single i.a. HA injection may thus represent the best balance between cure and treatment compliance.

Reports on the efficacy of i.a. HA injection on the treatment of people with an active lifestyle diagnosed with knee OA are still limited, and no studies have ever been carried out to assess the effectiveness of a Hymovis MO.RE. single-injection protocol in sportspeople. This study, therefore, aims to investigate, using clinical parameters and biomechanical assessments through GAIT analysis, the efficacy and safety of a single i.a. injection of Hymovis MO.RE. (32 mg/4 ml) as a treatment for adult regular sports players affected by knee OA.

## Material and Methods

This was a prospective, uncontrolled, mono-center, post-marketing study. Patients were recruited over a 6-month period and evaluated over the following 12 months (enrollment period May 2016-July 2018). The study was carried out at the Sapienza University, University Hospital Umberto I, Rome, Italy and was conducted in accordance with the Declaration of Helsinki and compliance with Good Clinical Practice (GCP) guidelines. All patients provided their informed consent, and the study protocol was approved by the Sapienza University Ethics Committee (Protocol Number EQL2-15–01, Approval No. 3997, Clinicaltrials.gov Identifier: NCT04661111).

### Inclusion and Exclusion Criteria

Patients were eligible for the study if they met all the following criteria: 1) age between 18 and 65 years; 2) an active lifestyle as a professional or regular sport player, i.e., training at least 2–3 times per week; 3) diagnosis of knee OA (classified as a Kellgren and Lawrence (KL) Grade I-III) (Kellgren and Lawrence, 1957) based on a standing weight bearing knee X-ray performed at screening or within 6 months prior to enrollment; 4) a knee visual analogue scale (VAS) pain score, performed within 48 h before the visit Day 0 ≥ 30 mm but ≤80 mm if receiving any analgesic medications; or a pain score of 40 –90 mm for patients free of any analgesic; the contralateral knee should have had a VAS pain score of <30 mm (the target knee was the one demonstrating the greatest VAS pain); 5) patients must have had all analgesic/anti-inflammatory drugs discontinued for 2 weeks prior to therapy except for acetaminophen; 6) the rescue medication, acetaminophen (maximum dose of 3 g/die), or any other analgesic, had not to be taken within 48 h of any visit. Patients were excluded from the study when either one or more clinically significant condition would interfere in the treatment and assessment of the study, or they underwent a prior surgical intervention involving the target joint, or had a history of allergic reaction to an i.a. HA injection or to acetaminophen, or they were participating in other trials, or had a case of previously identified knee trauma.

### Objectives and Endpoints

The primary study objective was to investigate if a single i.a. injection of Hymovis MO.RE. increased knee function 90 days after treatment. The primary endpoint was the average score of the SP1-SP5 questions of the “function during sport and recreational activity” subscale of the Knee injury and Osteoarthritis Outcome Score (KOOS) questionnaire (Roos and Lohmander, 2003). Score changes were normalized according to a 0–100 scale, increasing with patient improvements.

The secondary objectives of the study included further assessing the efficacy as well as safety of the single injection treatment with Hymovis MO.RE. 90 days after treatment. Additional efficacy objectives and endpoints included measuring biomechanical changes through GAIT analysis, the effect on pain perception through the VAS, WOMAC-A scores (Bellamy et al., 1988), and the KOOS pain subscale (Roos and Lohmander, 2003), again assessing the effect of treatment on knee function through the WOMAC-C score (Bellamy et al., 1988), and evaluating symptoms, activities of daily living (ADL) and quality of life (QOL) of patients using the corresponding KOOS subscores (Roos and Lohmander, 2003). The study also aimed to assess the requirements for daily rescue analgesic medications (simple analgesics, acetaminophen, the allowed “rescue dose” being acetaminophen 3 g/die) while being treated with Hymovis MO.RE.

GAIT analysis involved assessing mean velocity (m/s) and cadence (step/min) (Davis et al., 1991; Ornetti et al., 2010). The GAIT approach is a non-invasive method to measure body kinematics and kinetics: patients were requested to walk at a self-selected speed along a 10 m level surface after applying on their knee several retroreflective spherical markers to determine the joint centers and segment axis (Davis protocol) (Davis et al., 1991). The walk was repeated five times. GAIT data collection and analysis was performed using the ELITE system (BTS, Milano, Italy), with eight infrared video cameras (TVC, BTS, Milano, Italy) for the acquisition of the kinematic and kinetic variables. Two Kistler platforms (Kistler Instruments, Winterthur, Switzerland) were employed to acquire the ground reaction forces (GRF). (Paoloni et al., 2012; Alviti et al., 2017; Lo Torto et al., 2020). The VAS scale used in the present study was a standard 0–100 mm horizontal line, with 0 indicating no pain and 100 mm indicating the worst possible pain. The WOMAC A (5 questions) pain and the WOMAC C (12 questions) knee function questionnaires were as described by Bellamy and colleagues (1988). WOMAC scores were processed as described in the WOMAC user manual, that is, calculating a normalized score ranging from 0 (worst clinical condition) to 100 (best clinical condition). KOOS scores were also normalized, by calculating a 0 (worst condition) to 100 (best condition) KOOS index, as described in the 2012 KOOS user guide. Safety endpoints included the frequency and type of adverse events, patient withdrawals, and the presence of swelling, tenderness, crepitus, redness, and effusion at the target knee.

### Enrollment, Treatment, and Follow-up Visits

Patients were enrolled during a screening visit and underwent i.a. injection within 15 days, this visit was regarded as the study baseline. The injection was carried out through a lateral approach using a 4 ml-pre-filled syringe, corresponding to 32 mg Hymovis MO.RE. Following administration, passive flexion and extension of the knee were briefly performed to allow diffusion of the injected fluid throughout the joint. Patients had to limit movements during the following 24 h. Follow-up visits followed at 30, 60, 90, 135, 180, 270, and 360 days thereafter. Clinical (KOOS, VAS, WOMAC) scores were collected at baseline and at the 30, 90, 180, and 360-day control visits; biomechanical (GAIT) data were recorded at 30-, 90- and 180-days. Data concerning concomitant medications and adverse events were collected at each control visit. The 60, 135, and 270-day control visits were carried out by interviewing the patient over the phone and recording information about concomitant medications and adverse events.

### Data Analysis

The study sample size, 31 patients, was calculated based on the hypothesis of observing a minimum 15-point KOOS sport and recreational activity subscore (primary endpoint) change at 90 days after injection, compared to baseline, with a standard deviation of 21.1 points (Sakpal 2010), and 10% maximum possible dropout. Calculations were made by assuming that the results of statistical tests had 5% maximum error and 95% power. Categorical variables were reported as percentages; non-normally distributed variables were described by medians and ranges; normally distributed variables were described calculating their mean and standard deviation (SD). To investigate the primary and secondary objectives, *t*-tests for paired data or Wilcoxon signed-rank tests were used to compare VAS, WOMAC, and KOOS scores collected at each time point with those at baseline. To investigate how scores varied over time, they were also analyzed by means of parametric o non-parametric analysis of variance methods, if they had a normal or non-normal distribution respectively. Score changes were regarded as statistically significant if *p* < 0.05. All statistical analyses were performed using SPSS version 21.0 (IBM Clinical Software). Descriptive statistics were reported at each time for spatial-temporal gait parameters to evaluate the active functional assessment in patients with knee overuse syndrome. Data were expressed as the number of observations, mean, standard deviation (SD), and median. For each parameter, the comparison between treated and untreated knees was carried out using an analysis of the variance. Alternatively, a non-parametric approach has been proposed, the Wilcoxon Rank-Sum test. This test, based on data ranks, was used to compare GAIT parameters between two independent populations without the assumption of normally distributed data.

A General Linear Model (GLM) approach was also used to compare the difference from baseline, as a dependent variable, treatment group, as a factor, and baseline, as a covariate. Linear Regression analysis was used to evaluate the correlation between GAIT parameters and VAS or a knee-specific questionnaire, developed to assess the patients' opinion about their knee (KOOS, WOMAC-A). The Full Analysis Set (FAS) was used for all statistical analyses and data presentation. Considering that the outliers did not increase the valuable information, the analysis was repeated after removing the outliers from the dataset. The outliers were identified using the Explore procedure. To decide how to handle outliers, the first step used the source dataset and ensured that there were no data entries or tool errors. When a value was identified as an outlier, it was compared to the standardized value. The efficacy analysis was conducted with and without the outliers identified by the Explore procedure. Missing data were not replaced.

### Correlation Analysis

We described the relationship between clinical outcomes (scores) and spatio-temporal GAIT parameters, using a correlation analysis, which considers the entire patient cohort. In a linear model, we can judge how well the line fits the data (goodness of fit) by calculating the coefficient of determination (or square of the correlation coefficient) R2, which can be interpreted as the percentage of variance in the outcome variable that is explained by the predictor variable. Furthermore, in a linear regression analysis, it is important to evaluate the regression beta coefficient (β), which can be negative or positive and can have a t-value and a significance of the t-value, associated with each outcome. The β coefficient is the degree of change in the outcome variable for every 1-unit of change in the predictor variable. The *t*-test assesses whether the β coefficient is significantly different from zero. If the β coefficient is not statistically significant (i.e., the t-value is not significant), the variable does not significantly predict the outcome. If the β coefficient is significant, examine the sign of the β. If the β coefficient is positive, the interpretation is that for every 1-unit increase in the predictor variable, the outcome variable will increase by the β coefficient value. If the β coefficient is negative, the interpretation is that for every 1-unit increase in the predictor variable, the outcome variable will decrease by the β coefficient value.

## Results

### Patient’s Characteristics

Thirty-two subjects were screened, and 31 patients were enrolled. All were Caucasian, and 23 (74.2%) were women. Their median age was 49 years (range, 25–65). Twelve patients (38.7%) had Kellgren-Lawrence grade I, 16 (51.6%) grade II, and 3 (9.7%) grade III. Nineteen (61.3%) patients suffered from OA in the right knee, 12 (38.7%) in the left one. The VAS median value at the target knee was 72 mm (range, 43–87 mm) while that at the contralateral knee was 0 for all patients. At the screening visit, all patients were in good clinical status, including 5 (16.1%) who were receiving concomitant therapy for previous, not study-related, medical conditions (post bypass (*n* = 3), hypertension, flu). Twenty-three patients (74.2%) completed the study. Eight (25.8%) did not, either because they were lost to follow-up (*n* = 2) or withdrew informed consent (*n* = 6).

### Primary Endpoint

A statistically significant increase (*p* < 0.001) in the knee function KOOS score, compared to baseline, was detected 90 days after the i.a. injection, as well as at every other time point, confirming the efficacy of the treatment. The functional knee improvement increased through the entire follow-up period of interest (Table 1).

**TABLE 1:** Primary efficacy endpoint analysis. Statistical significance refers to comparison with baseline.

Parameter	Baseline (*n* = 31)		Day 30 (*n* = 31)		Day 90 (*n* = 29)		Day 180 (*n* = 26)		Day 360 (*n* = 23)	
	Mean ± SD	(Range)	Mean ± SD	(Range)	Mean ± SD	(Range)	Mean ± SD	(Range)	Mean ± SD	(Range)
KOOS–knee function^a^	41.610 ± 20.950	(15–95)	69.680 ± 24.930	(10–100)	74.660 ± 21.630	(15–100)	76.150 ± 18.460	(20–100)	78.040 ± 19.530	(20–100)
*p*-value			<0.001		<0.001		<0.001		<0.001	

a Knee function: knee functionality during sport and recreational activity (score); SD: standard deviation.

### Secondary Endpoints

**FIGURE 1:**
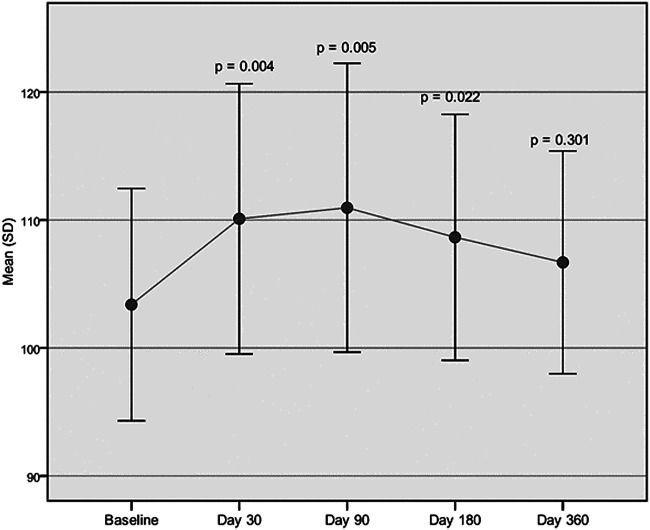
Cadence assessed through GAIT analysis. Mean cadence (step/min) at baseline compared to the mean cadence recorded at each follow-up visit.

**FIGURE 2:**
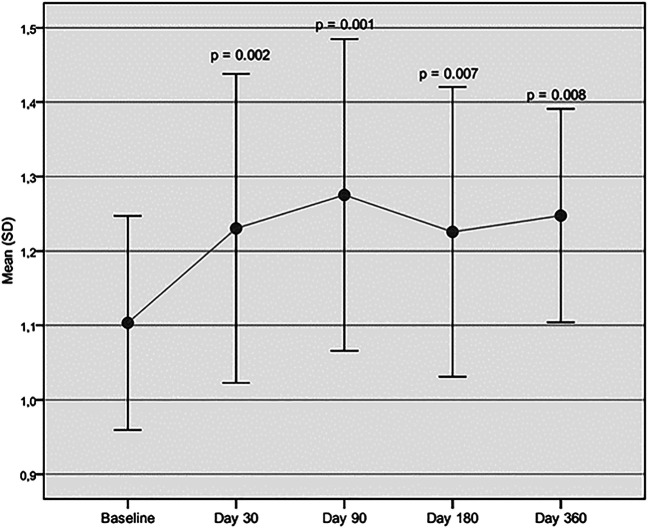
Velocity assessed through GAIT analysis. Mean velocity (m/s) at baseline is compared to mean velocity recorded at each follow-up visit.

Both cadence and velocity were assessed through GAIT analysis and showed a main improvement at 90 days after injection of Hymovis MO.RE. Cadence increased from the mean baseline value of 103.39 ± 9.07 to 110.95 ± 11.29 (*p* = 0.005). Significant statistical differences from baseline were observed even at day 30 and day 180 (Figure 1). Velocity increased from the baseline value of 1.10 ± 0.14 to 1.28 ± 0.21 (*p* = 0.001). Velocity was found to have significantly increased at every evaluation visit, even at day 360 (Figure 2). The VAS scores decreased significantly at all time points compared to baseline (*p* < 0.001 in all cases) (Table 2). Even the WOMAC-A pain score decreased significantly at all time points compared to baseline (*p* < 0.001) (Table 2). Difficulty in conducting normal activities related to knee function decreased significantly at all time points (*p* < 0.001 in all cases) (Table 2).

**TABLE 2:** Secondary endpoints analysis. Statistical significance refers to comparison with baseline.

Parameter	Baseline (*n* = 31)		Day 30 (*n* = 31)		Day 90 (*n* = 29)		Day 180 (*n* = 26)		Day 360 (*n* = 23)	
	Mean ± SD	(Range)	Mean ± SD	(Range)	Mean ± SD	(Range)	Mean ± SD	(Range)	Mean ± SD	(Range)
VAS-knee pain^a^	68.650 ± 11.530	(42–86)	20.940 ± 19.100	(0–86)	16.480 ± 18.650	(0–84)	12.150 ± 17.290	(0–75)	14.390 ± 15.410	(0–70)
*p*-value			<0.001		<0.001		<0.001		<0.001	
WOMAC-A-knee pain^a^	27.740 ± 13.840	(5–55)	11.770 ± 10.530	(0–35)	7.410 ± 9.320	(0–30)	6.200 ± 11.410	(0–35)	8.040 ± 9.620	(0–35)
*p*-value			<0.001		<0.001		<0.001		<0.001	
WOMAC-C–knee function^a^	23.580 ± 15.950	(0–52.940)	9.290 ± 10.330	(0–35.290)	7.200 ± 9.520	(0–33.820)	6.450 ± 12.130	(0–47.060)	5.050 ± 10.000	(0–47.060)
*p*-value			<0.001		<0.001		<0.001		<0.001	
KOOS–Knee pain^a^	70.070 ± 13.240	(41.670–88.890)	85.660 ± 11.890	(55.560–100)	87.640 ± 10.540	(63.890–100)	90.280 ± 10.340	(63.890–100)	88.160 ± 9.780	(58.330–100)
*p*-value			<0.001		<0.001		<0.001		<0.001	
KOOS–Symptoms^a^	60.650 ± 23.790	(5–95)	78.710 ± 17.890	(30–100)	83.280 ± 14.840	(45–100)	83.650 ± 15.330	(40–100)	83.480 ± 17.090	(35–100)
*p*-value			<0.001		<0.001		<0.001		<0.001	
KOOS–Activity of daily living^a^	79.930 ± 14.380	(48.530–100)	92.170 ± 9.750	(64.710–100)	93.970 ± 9.190	(63.240–100)	94.910 ± 9.170	(61.760–100)	95.650 ± 7.690	(64.710–100)
*p*-value			<0.001		<0.001		<0.001		<0.001	
KOOS–Quality of life^a^	45.160 ± 18.520	(6.250–93.750)	61.090 ± 27.290	0–100)	66.160 ± 24.460	(6.250–100)	68.270 ± 23.450	(0–100)	68.210 ± 23.830	(0–100)
*p*-value			0.001		<0.001		<0.001		<0.001	

SD: standard deviation.

a score.

Pain, symptoms, and QOL measured by the corresponding KOOS subscores showed a statistically significant improvement, compared to baseline, at all-time points (*p* < 0.001). The subjective assessment of ADL, measured by the ADL KOOS subscore, also improved significantly compared to baseline at all time points (*p* < 0.001), (Table 2).

**FIGURE 3:**
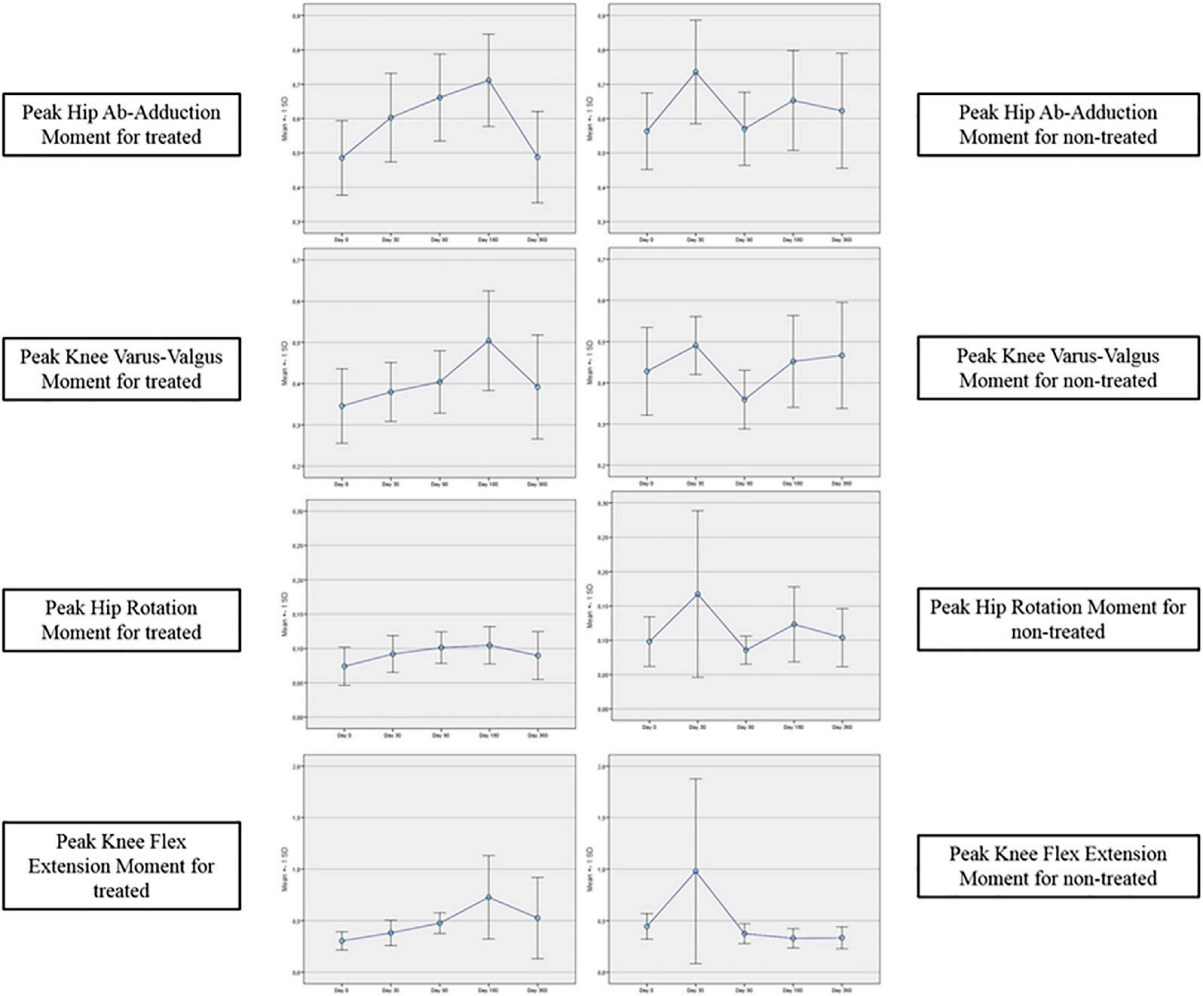
GAIT parameters grouped by treatment (full cohort).

Tables 3A–D reported the descriptive statistics at each study time point and the results of statistical comparisons, considering the full cohort, between the changes from baseline (Day 0) for the GAIT parameters grouped by treatment. In particular, a statistical significance with a *p*-value < 0.05 in the comparison between the two groups “Treated knee” vs. “No-Treated” has been detected for the following parameters: Peak Hip Ab-Adduction Moment, Peak Knee Valgus-Varus Moment, Peak Hip Rotation Moment, Peak of Knee Flex-Extension Moment (Figure 3).

**TABLE 3A:** (part 1 of 4). GAIT parameters full cohort. Peak Hip Ab-Adduction Moment. General Linear Model with Peak Hip Ab-Adduction Moment change from baseline as dependent variable, treatment as factor, and Peak Hip Ab-Adduction Moment baseline value as covariate.

Group		Baseline (Day 0)	Change from baseline			
			Day 30	Day 90	Day 180	Day 360
Treated	N	31	31	29	25	22
	Mean ± Standard Deviation	0.490 **±** 0.296	0.120 **±** 0.392	0.190 **±** 0.383	0.220 **±** 0.353	0.050 **±** 0.398
	Median	0.480	0.120	0.190	0.140	0.150
No treated	N	31	31	30	26	21
	Mean ± Standard Deviation	0.560b **±** 0.304	0.170 **±** 0.471	0.000 **±** 0.341	0.140 **±** 0.521	0.070 **±** 0.444
	Median	0.540	0.050	−0.010	0.170	−0.040
Total	N	62	62	59	51	43
	Mean ± Standard Deviation	0.520 **±** 0.300	0.140 **±** 0.431	0.090 **±** 0.372	0.180 **±** 0.444	0.060 **±** 0.416
	Median	0.520	0.110	0.020	0.15	0.13
ANOVA (*p*-value)		0.310	0.621	0.044	0.502	0.911
Wilcoxon (*p*-value)		0.356	0.714	0.061	0.624	0.817
GLM^1^ (*p*-value)		−	0.252	0.127	0.550	0.254

**TABLE 3B:** (part 2 of 4). GAIT parameters full cohort. Peak Knee Valgus-Varus Moment. General Linear Model with Peak Knee Valgus-Varus Moment change from baseline as dependent variable, treatment as factor, and Peak Knee Valgus-Varus Moment baseline value as covariate.

Group		Baseline (Day 0)	Change from baseline			
			Day 30	Day 90	Day 180	Day 360
Treated	N	31	31	29	25	22
	Mean ± Standard Deviation	0.350 **±** 0.246	0.030 **±** 0.250	0.080 **±** 0.267	0.160 **±** 0.269	0.080 **±** 0.298
	Median	0.350	0.030	0.090	0.190	0.160
No treated	N	31	31	30	26	21
	Mean ± Standard Deviation	0.430 **±** 0.290	0.060 **±** 0.341	−0.070 **±** 0.285	0.050 **±** 0.242	0.100 **±** 0.360
	Median	0.410	0.090	-0.070	0.050	0.040
Total	N	62	62	59	51	43
	Mean ± Standard Deviation	0.390 **±** 0.270	0.050 **±** 0.297	0.010 **±** 0.284	0.100 **±** 0.260	0.090 **±** 0.326
	Median	0.390	0.040	0.020	0.120	0.100
ANOVA (*p*-value)		0.235	0.707	0.039	0.112	0.860
Wilcoxon (*p*-value)		0.221	0.536	0.068	0.068	0.990
GLM^1^ (*p*-value)		−	0.048	0.169	0.175	0.473

**TABLE 3C:** (part 3 of 4). GAIT parameters full cohort. Peak Hip Rotation Moment. General Linear Model with Peak Hip Rotation Moment change from baseline as dependent variable, treatment as factor, and Peak Hip Rotation Moment baseline value as covariate.

Group		Baseline (Day 0)	Change from baseline			
			Day 30	Day 90	Day 180	Day 360
Treated	N	31	31	29	25	22
	Mean ± Standard Deviation	0.070 **±** 0.76	0.020 **±** 0.110	0.040 **±** 0.070	0.040 **±** 0.074	0.030 **±** 0.092
	Median	0.060	0.020	0.040	0.030	0.030
No treated	N	31	31	30	26	21
	Mean ± Standard Deviation	0.100 **±** 0.098	0.070 **±** 0.339	−0.010 **±** 0.111	0.050 **±** 0.157	0.020 **±** 0.105
	Median	0.070	0.030	−0.010	0.000	0.020
Total	N	62	62	59	51	43
	Mean ± Standard Deviation	0.090 **±** 0.088	0.040 **±** 0.251	0.010 **±** 0.096	0.040 **±** 0.122	0.030 **±** 0.098
	Median	0.070	0.020	0.020	0.020	0.020
ANOVA (*p*-value)		0.285	0.426	0.035	0.907	0.694
Wilcoxon (*p*-value)		0.155	0.811	0.039	0.147	0.610
GLM^1^ (*p*-value)		−	0.242	0.254	0.453	0.533

**TABLE 3D:** (part 4 of 4). GAIT parameters full cohort. Peak Knee Flex-Extension Moment. General Linear Model with Peak Knee Flex Extension Moment change from baseline as dependent variable, treatment as factor, and Peak Knee Flex Extension Moment baseline value as covariate.

Group		Baseline (Day 0)	Change from baseline			
			Day 30	Day 90	Day 180	Day 360
Treated	N	31	31	29	25	22
	Mean ± standard Deviation	0.300 **±** 0.242	0.080 **±** 0.380	0.160 **±** 0.360	0.390 **±** 1.067	0.190 **±** 0.970
	Median	0.250	0.060	0.130	0.230	-0.020
No treated	N	31	31	30	26	21
	Mean ± standard Deviation	0.440 **±** 0.340	0.540 **±** 2.377	−0.060 **±** 0.426	−0.110 **±** 0.460	−0.100 **±** 0.399
	Median	0.370	0.040	−0.090	0.000	−0.140
Total	N	62	62	59	51	43
	Mean ± standard Deviation	0.370 **±** 0.301	0.310 **±** 1.704	0.050 **±** 0.407	0.130 **±** 0.847	0.050 **±** 0.754
	Median	0.300	0.060	0.060	0.070	-0.070
ANOVA (*p*-value)		0.065	0.294	0.038	0.032	0.205
Wilcoxon (*p*-value)		0.091	0.778	0.054	0.018	0.224
GLM^1^ (*p*-value)		−	0.368	0.148	0.079	0.433

**FIGURE 4:**
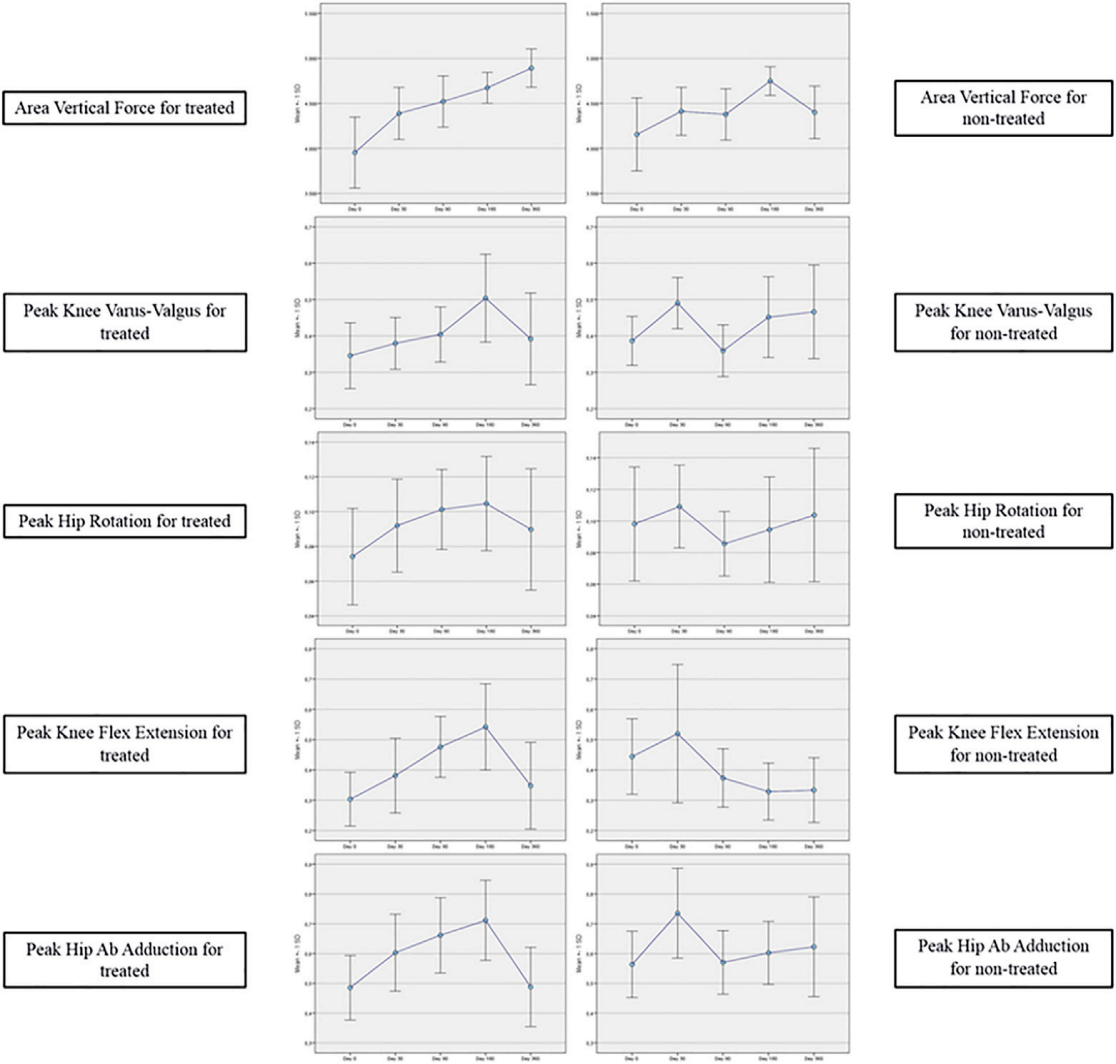
GAIT parameters grouped by treatment (cohort without outliers).

Tables 4A–E reported the descriptive statistics at each study time point and the results of statistical comparisons, considering the cohort without outliers, between the changes from baseline (Day 0) for the GAIT parameters grouped by treatment. In particular, a statistical significance with a *p*-value < 0.05 in the comparison between the two groups “Treated knee” vs. “No-Treated Knee” has been detected for the following parameters: Vertical Force Area, Peak Knee Valgus-Varus, Peak Hip Rotation, Peak Knee Flex-Extension and Peak Hip Adduction (Figure 4).

**TABLE 4A:** (part 1 of 5). GAIT parameters cohort without outliers. Area Vertical Force. General Linear Model with Area Vertical Force change from baseline as dependent variable, treatment as factor, and Area Vertical Force baseline value as covariate.

Group		Baseline (Day 0)	Change from baseline			
			Day 30	Day 90	Day 180	Day 360
Treated	N	28	26	24	21	12
	Mean ± Standard Deviation	3952.500 **±** 1017.146	425.340 **±** 1211.849	645.610 **±** 1407.237	876.290 **±** 1105.504	941.690 **±** 701.915
	Median	4274.140	260.830	772.700	596.410	1149.860
No treated	N	29	27	25	22	16
	Mean ± Standard Deviation	4153.860 **±** 1067.160	193.030 **±** 1423.090	241.570 **±** 1372.066	685.190 **±** 1294.663	29.090 **±** 1039.353
	Median	4460.260	−62.630	150.970	443.940	108.640
Total	N	57	53	49	43	28
	Mean ± Standard Deviation	4054.940 **±** 1038.532	307.000 **±** 1316.202	439.470 **±** 1389.903	778.520 **±** 1195.571	420.200 **±** 1006.171
	Median	4389.150	137.710	380.260	586.720	573.000
ANOVA (*p*-value)		0.469	0.526	0.314	0.606	0.014
Wilcoxon (*p*-value)		0.202	0.434	0.254	0.437	0.014
GLM^1^ (*p*-value)		−	0.974	0.422	0.470	0.010

**TABLE 4B:** (part 2 of 5). GAIT parameters cohort without outliers. Peak Knee Valgus-Varus. General Linear Model with Peak Knee Valgus-Varus change from baseline as dependent variable, treatment as factor, and Peak Knee Valgus-Varus baseline value as covariate.

Group		Baseline (Day 0)	Change from baseline			
			Day 30	Day 90	Day 180	Day 360
Treated	N	31	31	29	25	22
	Mean ± standard Deviation	0.350 **±** 0.246	0.030 **±** 0.250	0.080 **±** 0.267	0.160 **±** 0.269	0.080 **±** 0.298
	Median	0.350	0.030	0.090	0.190	0.160
No treated	N	30	30	29	25	21
	Mean ± standard Deviation	0.390 **±** 0.180	0.100 **±** 0.252	−0.040 **±** 0.233	0.060 **±** 0.237	0.100 **±** 0.360
	Median	0.410	0.100	−0.070	0.080	0.040
Total	N	61	61	58	50	43
	Mean ± standard Deviation	0.370 **±** 0.216	0.070 **±** 0.252	0.020 **±** 0.256	0.110 **±** 0.257	0.090 **±** 0.326
	Median	0.380	0.060	0.020	0.130	0.100
ANOVA (*p*-value)		0.465	0.275	0.072	0.159	0.860
Wilcoxon (*p*-value)		0.302	0.391	0.099	0.093	0.990
GLM^1^ (*p*-value)		−	0.041	0.172	0.151	0.473

**TABLE 4C:** (part 3 of 5). GAIT parameters cohort without outliers. Peak Hip Rotation. General Linear Model with Peak Hip Rotation change from baseline as dependent variable, treatment as factor, and Peak Hip Rotation baseline value as covariate.

Group		Baseline (Day 0)	Change from baseline			
			Day 30	Day 90	Day 180	Day 360
Treated	N	31	31	29	25	22
	Mean ± Standard Deviation	0.070 **±** 0.076	0.020 **±** 0.110	0.040 **±** 0.070	0.040 **±** 0.074	0.030 **±** 0.092
	Median	0.060	0.020	0.040	0.030	0.030
No treated	N	31	30	30	24	21
	Mean ± Standard Deviation	0.100 **±** 0.098	0.010 **±** 0.123	−0.010 **±** 0.111	0.010 **±** 0.105	0.020 **±** 0.105
	Median	0.070	0.030	−0.010	0.000	0.020
Total	N	62	61	59	49	43
	Mean ± Standard Deviation	0.090 **±** 0.088	0.020 **±** 0.116	0.010 **±** 0.096	0.030 **±** 0.091	0.030 **±** 0.098
	Median	0.070	0.020	0.020	0.010	0.020
ANOVA (*p*-value)		0.285	0.854	0.035	0.296	0.694
Wilcoxon (*p*-value)		0.155	0.983	0.039	0.041	0.610
GLM^1^ (*p*-value)		−	0.300	0.036	0.302	0.860

**TABLE 4D:** (part 4 of 5). GAIT parameters cohort without outliers. Peak Knee Flex-Extension. General Linear Model with Peak Knee Flex-Extension change from baseline as dependent variable, treatment as factor, and Peak Knee Flex-Extension baseline value as covariate.

Group		Baseline (Day 0)	Change from baseline			
			Day 30	Day 90	Day 180	Day 360
Treated	N	31	31	29	24	21
	Mean ± Standard Deviation	0.300 **±** 0.242	0.080 **±** 0.380	0.160 **±** 0.360	0.200 **±** 0.458	0.000 **±** 0.363
	Median	0.250	0.060	0.130	0.200	-0.040
No treated	N	31	29	30	26	21
	Mean ± Standard Deviation	0.440 **±** 0.340	0.080 **±** 0.550	−0.060 **±** 0.426	−0.110 **±** 0.460	−0.100 **±** 0.399
	Median	0.370	0.030	−0.090	0.000	−0.140
Total	N	62	60	59	50	42
	Mean ± Standard Deviation	0.370 **±** 0.301	0.080 **±** 0.466	0.050 **±** 0.407	0.040 **±** 0.481	−0.050 **±** 0.380
	Median	0.300	0.050	0.060	0.070	−0.070
ANOVA (*p*-value)		0.065	0.996	0.038	0.021	0.395
Wilcoxon (*p*-value)		0.091	0.451	0.054	0.028	0.320
GLM^1^ (*p*-value)		−	0.626	0.148	0.020	0.817

**TABLE 4E:** (part 5 of 5). GAIT parameters cohort without outliers. Peak Hip Ab-Adduction. General Linear Model with Peak Hip Ab-Adduction change from baseline as dependent variable, treatment as factor, and Peak Hip Ab-Adduction baseline value as covariate.

Group		Baseline (Day 0)	Change from baseline			
			Day 30	Day 90	Day 180	Day 360
Treated	N	31	31	29	25	22
	Mean ± Standard Deviation	0.490 **±** 0.296	0.120 **±** 0.392	0.190 **±** 0.383	0.220 **±** 0.353	0.050 **±** 0.398
	Median	0.480	0.120	0.190	0.140	0.150
No treated	N	31	31	30	25	21
	Mean ± Standard Deviation	0.560 **±** 0.304	0.170 **±** 0.471	0.000 **±** 0.341	0.080 **±** 0.427	0.070 **±** 0.444
	Median	0.540	0.050	−0.010	0.160	−0.040
Total	N	62	62	59	50	43
	Mean ± Standard Deviation	0.520 **±** 0.300	0.140 **±** 0.431	0.090 **±** 0.372	0.150 **±** 0.395	0.060 **±** 0.416
	Median	0.520	0.110	0.020	0.140	0.130
ANOVA (*p*-value)		0.310	0.621	0.044	0.196	0.911
Wilcoxon (*p*-value)		0.356	0.714	0.061	0.455	0.817
GLM^1^ (*p*-value)		−	0.252	0.127	0.187	0.254

### Results of Correlation Analysis

**FIGURE 5:**
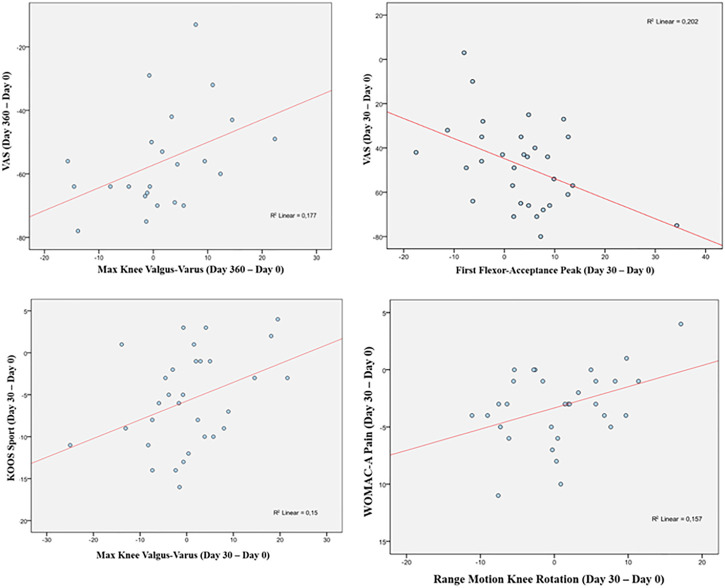
Correlation analysis.

**FIGURE 6:**
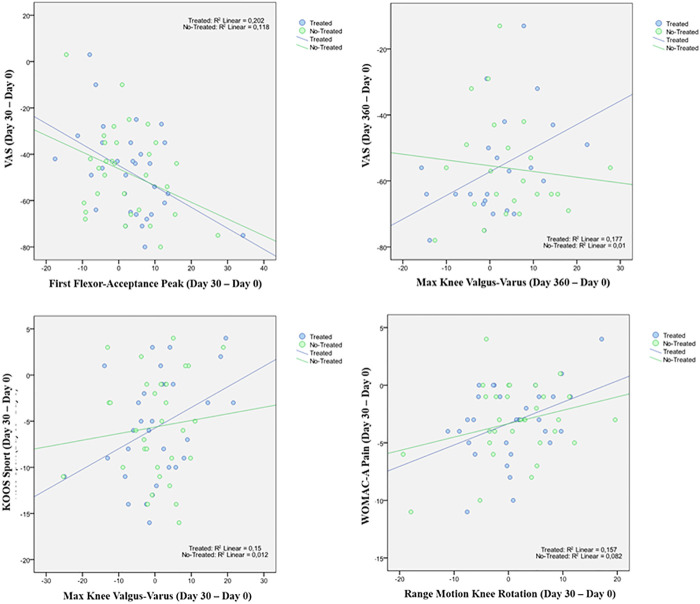
Linear Regression model.

A statistically significant correlation between “clinical outcome” vs. “GAIT parameter” has been detected in the “Knee treated” group for the following parameters (Table 5). For the predicted parameter “First Flexor-Acceptance Peak” vs. the outcome variable “VAS” the R2 is 0.202, i.e., around 20% of the variance in the outcome is explained by the predictor, the β coefficient is negative i.e., for every 1-unit increase in the “First Flexor-Acceptance Peak Day 30 change from Baseline”, the “VAS Day 30 change from baseline” will decrease by the beta coefficient value (−0.905), with a *p*-value of 0.011; for the “Max Knee Valgus-Varus” vs. “VAS” the R2 is 0.177, i.e., around 2% of the variance in the outcome is explained by the predictor, the β coefficient is positive i.e., for every 1-unit increase in the “Max Knee Valgus-Varus Day 360 change from Baseline”, the “VAS Day 360 change from baseline” will increase by the beta coefficient value (0.717), with a *p*-value of 0.046; for the “Max Knee Valgus-Varus” vs. “KOOS-SPORT” the R2 is 0.15, i.e., around 1.5% of the variance in the outcome is explained by the predictor, the β coefficient is positive i.e., for every 1-unit increase in the “Max Knee Valgus-Varus Day 30 change from Baseline”, the “KOOS-SPORT Day 30 change from baseline” will increase by the beta coefficient value (0.223), with a *p*-value of 0.032; finally, for the “Range Motion Knee Rotation” vs. “WOMAC-A Pain” the R2 is 0.16, i.e., around 2% of the variance in the outcome is explained by the predictor, the β coefficient is positive i.e., for every 1-unit increase in the “Range Motion Knee Rotation Day 30 change from Baseline”, the “WOMAC-A Pain Day 30 change from baseline” will increase by the beta coefficient value (0.186), with a *p*-value of 0.028 (Figure 5). Moreover, we conducted an ANOVA analysis to determine if there is a statistical significance difference among the two groups “Treated Knee” vs. “Non-Treated Knee” in the four linear models inspected with the previous correlation analysis (Table 6). We noticed particularly interesting results because a significative correlation in the group “Treated” corresponds to a non-significative (or a non-correlation) in the other group “Non-Treated”. In particular, for the predictor “First Flexor-Acceptance Peak” and the outcome variable “VAS” grouped by treatment, the R2 is 0.202 (for “Treated” group) with a *p*-value of 0.011 and the R2 is 0.118 (for “Non-Treated” group) with a *p*-value of 0.059; for the predictor “Max Knee Valgus-Varus” and the outcome variable “VAS” grouped by treatment, the R2 is 0.177 (for “Treated” group) with a *p*-value of 0.046 and the R2 is 0.01 (for “Non-Treated” group) with a *p*-value of 0.645; for the predictor “Max Knee Valgus-Varus” and the outcome variable “KOOS-SPORT” grouped by treatment, the R2 is 0.15 (for “Treated” group) with a *p*-value of 0.032 and the R2 is 0.012 (for “Non-Treated” group) with a *p*-value of 0.561; finally, for the predictor “Range Motion Knee Rotation” and the outcome variable “WOMAC-A PAIN” grouped by treatment, the R2 is 0.157 (for “Treated” group) with a *p*-value of 0.028 and the R2 is 0.082 (for “Non-Treated” group) with a *p*-value of 0.118. No severe adverse events (SAE) were reported during i.a. injections or in the follow-up period. Six (19%) patients experienced six adverse events ranging from mild to moderate, all of which were considered unrelated to the investigational product. Two patients had flu, two patients had road accidents, one experienced abdominal pain and one had sternoclavicular pain. All patients who experienced adverse events recovered completely, except one who was lost at the follow-up. Acetaminophen was administered as rescue medication to three patients during treatment with a median daily dose of 1,000 mg for 2, 8, and 15 days, respectively, (Figure 6).

**TABLE 5:** Correlation analysis.

Parameters	Knee	Time (day)	β	*p*-value
First Flexor-Acceptance Peak vs. VAS	Treated	30	−0.905	**0.011**
Max Knee Valgus-Varus vs. VAS	Treated	360	0.717	**0.046**
Max Knee Valgus-Varus vs. KOOS-Sport	Treated	30	0.223	**0.032**
Range Motion Knee Rotation vs. WOMAC-A Pain	Treated	30	0.186	**0.028**

**TABLE 6:** ANOVA.

First Flexor-Acceptance Peak vs. VAS by treatment group (Day 30–Day 0)					
Knee	Sum of squares	df	Mean square	F	Sig
Treated	2253.788	1	2253.788	7.360	0.011
No-treated	1311.226	1	1311.226	3.871	0.059
Max Knee Valgus-Varus vs. VAS by treatment group (Day 360–Day 0)					
Treated	975.872	1	975.872	4.517	0.046
No-treated	56.772	1	56.772	0.219	0.645
Max Knee Valgus-Varus vs. KOOS-SPORT by treatment group (Day 30–Day 0)					
Treated	143.865	1	143.865	5.104	0.032
No-treated	11.357	1	11.357	0.347	0.561
Range Motion Knee Rotation vs. WOMAC-A Pain by treatment group (Day 30–Day 0)					
Treated	48.380	1	48.380	5.387	0.028
No-treated	25.379	1	25.379	2.596	0.118

## Discussion

The results of the present study show that a single Hymovis MO.RE. injection at the selected dosage (32 mg/4 ml) is safe and effective for treating knee OA in sports players between 18 and 65 years and good clinical status. The main clinical improvement consisted in a significant increase of knee function, as measured by the KOOS scores, starting at day 30 and lasting over one year from treatment, according to a pattern confirmed by similar changes of the WOMAC-C score over time. The robustness of these findings is enhanced by the results of concomitant GAIT evaluations, which were consistent as they exhibited the same variation pattern of the KOOS function and WOMAC-C subjective scores: mean cadence and velocity significantly increased from the first follow-up visit (day 30). GAIT measurements can be regarded as objective and sensitive, as they are influenced by the knee OA status only (Ornetti et al., 2010) and allow to distinguish healthy persons from those affected by lower limb OA as well as mild knee OA from severe one (Zeni and Higginson, 2009). Results of the present study also show that a single Hymovis MO.RE. i.a. injection is effective in decreasing pain over time, providing an early and lasting benefit, as shown by changes of all three pain scores used in the present study, i.e., the VAS, WOMAC-A, and KOOS pain scores, all showing the same improvement pattern. Improvement in function and symptoms led the patients to experience a significant improvement in ADL and QOL, in 30 days only. A single Hymovis MO.RE. injection, thus, may allow rapid and lasting benefits in sportsmen affected by KL grade I-III knee also concerning their overall perception of their quality of life over their daily activity.

The results of the present study reflect those obtained through multiple Hymovis injections at different dosages (Benazzo et al., 2016; Henrotin et al., 2019). Henrotin et al. observed similar results concerning KOOS scores, in 46 knee OA patients treated with two Hymovis (8 mg/3 ml) injection cycles carried out 6 months apart, each cycle consisting of two injections, performed one week apart (Henrotin et al., 2019). Benazzo and colleagues (2016) observed a long-lasting response to treatment in a prospective study involving 50 knee OA patients receiving two Hymovis (8 mg/3 ml) injections one week apart. At the 3-month follow-up, the total WOMAC score decreased significantly compared to baseline, and the percentage of patients experiencing moderate pain, as measured by the WOMAC-A score, was 19% after one year, compared to 80% at baseline. In that study, forty-two patients (84%) were younger than 50, making the study cohort comparable to that of the present work. However, these studies should be compared with caution, because they differ in some patients’ characteristics at baseline, including their median age and physical activity. Yet, all seem to indicate that a single 32 mg/4 ml Hymovis MO.RE. i. a. injection reduces pain and enhances knee functionality in patients with an active lifestyle as effectively as a repetitive injection at a lower volume. Confirming this would be clinically relevant for any patients, and especially for sports players, because they would benefit from several advantages, namely that a single injection 1) is a rapid and minimally invasive procedure, with a reduced recovery time from treatment and allowing early return to training; 2) its administration can be arranged taking into consideration the patients’ needs related to training sessions; 3) it represents a viable treatment option, as sportsmen expect rapid resolution of symptoms, fast recovery, and are usually little compliant to long-term therapies. A single dose schedule may also lower the risk of adverse events, such as joint swelling and arthralgia (Concoff et al., 2017; Altman et al., 2018), making the treatment more attractive, and increasing compliance.

Further advantages of a single injection involve reducing the treatment burden and costs for patients (McElheny et al., 2019), including time away from work or training because of the need for fewer transfers to medical facilities and reduced recovery time. The procedure, requiring 3-day of rest, represents a chance for regular sports players who need to keep absence from sports activity to a minimum. The single Hymovis MO.RE. i.a. injection showing long-lasting efficacy and tolerability also implies that this single dosing regimen may be repeated once a year, becoming a long-term treatment that could delay surgical knee intervention (Migliore and Procopio, 2015). This should be the subject of appropriately designed future prospective investigations.

The positive effect of Hymovis MO.RE. on function and pain observed in the present study are consistent with its molecular reticulum having the capacity to continuously recover its 3-dimensional configuration, even after intense and repetitive mechanical stress, making it an ideal shock absorber (Finelli et al., 2011; Migliore and Procopio, 2015). Furthermore, prospective comparative studies should be carried out to assess other HA-based formulations with a similar molecular weight, but different rheological properties, in comparison with Hymovis MO.RE. when administered by a single i.a. injection, in sportsmen as well as in other patient subgroups.

In the analysis of statistical comparisons between the baseline (Day 0) and each study data point for the GAIT parameters grouped by treatment, we noticed a statistical significance, with a *p*-value < 0.05, in the comparison between the two groups “Treated knee” vs. “No-Treated” for the following parameters: Peak Hip Ab-Adduction Moment, Peak Knee Valgus-Varus Moment, Peak Hip Rotation Moment, Peak Knee Flex-Extension Moment. We also reported the descriptive statistics at each study time point and the results of statistical comparisons, considering the cohort without outliers, between the changes from baseline (Day 0) for the GAIT parameters grouped by treatment. In particular, a statistical significance, with a *p*-value < 0.05, in the comparison between the two groups “Treated knee” vs. “No-Treated Knee” has been detected for the following parameters: Vertical Force Area, Peak Knee Valgus-Varus, Peak Hip Rotation, Peak Knee Flex-Extension, and Peak Hip Adduction. Moreover, we described the relationship between clinical outcomes (scores) and biochemical parameters (obtained by GAIT analysis), using correlation analysis, considering the whole cohort of patients. A statistically significative correlation between “clinical outcome” vs. “GAIT parameter” has been detected in the “Knee treated” group for the following parameters: “First Flexor-Acceptance Peak” vs. “VAS”, “Max Knee Valgus-Varus” vs. “VAS”, “Max Knee Valgus-Varus” vs. “KOOS-SPORT” and “Range Motion Knee Rotation” vs. “WOMAC-A Pain”.

We also conducted an ANOVA analysis to determine if there is a statistically significant difference between the two groups, “Treated Knee” vs. “Non-Treated Knee”, in the four linear models inspected with the previous correlation analysis. We observed a significative correlation in the “Treated” group corresponds to a no-significative (or a no-correlation) in the other “No-Treated” group for this comparisons: for the predictor “First Flexor-Acceptance Peak” and the outcome variable “VAS”; for the predictor “Max Knee Valgus-Varus” and the outcome variable “VAS”; for the predictor “Max Knee Valgus-Varus” and the outcome variable “KOOS-SPORT”; finally, for the predictor “Range Motion Knee Rotation” and the outcome variable “WOMAC-A PAIN”.

The results of this study are interesting and innovative in light of the international literature relating to the impact of the therapy with the Hymovis SPORT intra-articular injection therapy in patients affected by knee overuse syndrome. The results of the present work also show that Hymovis MO.RE. has a good safety profile, since adverse events were not treatment-related, and no SAEs were detected. This further supports Hymovis MO.RE. injection as a viable therapy, possibly increasing patient compliance to treatment.

### Study Limitations

The main limitation of the present study is its small sample size and the lack of a similar control group undergoing the standard treatment. Moreover, only three patients had a grade III KL OA and a surface EMG was not used. Furthermore, no specific questionnaires exploring sports performance were administered to patients, thus allowing only for an indirect assessment of effects specifically concerning sports activity. This calls for large prospective cohort studies to further assess the efficacy of this treatment in regular sports players affected by knee OA. Given the clinical characteristics and expectations of this particular patient population, future studies are also advised to evaluate the possible preventive role of i. a. Hymovis MO.RE. injection in reducing knee injuries as well as its effectiveness when associated with physical rehabilitation. Further investigations should include GAIT assessment, given it may provide objective confirmation of subjectively-measured scores, and it has been shown to be predictive of OA progression (Favre and Jolles, 2017).

## Conclusion

The results of this study represent a step forward in the management of gonarthrosis pain and the return to sports activity. A single Hymovis MO.RE. (32 mg/4 ml) intra-articular injection seems to provide a rapid, lasting, and safe response in regular sports players affected by knee OA. Treatment with Hymovis MO.RE. may therefore be a viable therapeutic option that meets the needs of the demanding subgroup of active lifestyle patients affected by knee OA arising from overuse injuries. Extensive investigations, including a control group, should be conducted in the future to confirm the efficacy of this single dose regimen.

## Data Availability

The original contributions presented in the study are included in the article/Supplementary Material, further inquiries can be directed to the corresponding author.
